# Evaluation of ENSO simulations in CMIP5 models: A new perspective based on percolation phase transition in complex networks

**DOI:** 10.1038/s41598-018-33340-y

**Published:** 2018-10-08

**Authors:** Zhenghui Lu, Zuntao Fu, Lijuan Hua, Naiming Yuan, Lin Chen

**Affiliations:** 1 CAS Key Laboratory of Regional Climate-Environment for Temperate East Asia, Institute of Atmospheric Physics, Chinese Academy of Sciences, 100029 Beijing, China; 2 Lab for Climate and Ocean-Atmosphere Studies, Dept. of Atmospheric and Oceanic Sciences, School of Physics, Peking University, Beijing, 100871 China; 3 State Key Laboratory of Severe Weather (LASW), Chinese Academy of Meteorological Sciences, Beijing, 100081 China; 4 International Pacific Research Center, and School of Ocean and Earth Science and Technology, University of Hawaii at Manoa, Honolulu, Hawaii USA

## Abstract

In this study, the performance of CMIP5 models in simulating the El Niño-Southern Oscillation (ENSO) is evaluated by using a new metric based on percolation theory. The surface air temperatures (SATs) over the tropical Pacific Ocean are constructed as a SAT network, and the nodes within the network are linked if they are highly connected (e.g., high correlations). It has been confirmed from reanalysis datasets that the SAT network undergoes an abrupt percolation phase transition when the influences of the sea surface temperature anomalies (SSTAs) below are strong enough. However, from simulations of the CMIP5 models, most models are found incapable of capturing the observed phase transition at a proper critical point *P*_*c*_. For the 15 considered models, four even miss the phase transition, indicating that the simulated SAT network is too stable to be significantly changed by the SSTA below. Only four models can be considered cautiously with some skills in simulating the observed phase transition of the SAT network. By comparing the simulated SSTA patterns with the node vulnerabilities, which is the chance of each node being isolated during a ENSO event, we find that the improperly simulated sea-air interactions are responsible for the missing of the observed percolation phase transition. Accordingly, a careful study of the sea-air couplers, as well as the atmospheric components of the CMIP5 models is suggested. Since the percolation phase transition of the SAT network is a useful phenomenon to indicate whether the ENSO impacts can be transferred remotely, it deserves more attention for future model development.

## Introduction

El Niño-Southern Oscillation (ENSO), the dominant mode of inter-annual climate variability, is one of the most important ocean-atmosphere coupled phenomena. It is characterized by large-scale anomalous sea surface temperature (SST) warming (El Niño)/cooling (La Niña) in the tropical central eastern Pacific, and exerts great influences on regional and global climate^1–6^. As one of the most important natural phenomena, ENSO is an important indicator for the evaluation of Coupled General Circulation Models (CGCMs)^7–10^. During the past several decades, although ENSO simulation has been improved considerably, it is still challenging to fully capture all of its behaviors^7^. To evaluate the ENSO simulations in CGCMs, different metrics have been proposed in the past^7–11^. Some metrics concentrate on the basic ENSO features, such as ENSO amplitude (Niño3 SST standard deviation), frequency (root-mean-square error (RMSE) of Niño3 SSTA spectra), while some others evaluate relevant atmospheric processes such as atmospheric Bjerknes feedback^12^, which is computed as the linear regression coefficient between Niño4 zonal surface wind stress anomaly and Niño3 SST monthly anomaly.

With these metrics, many CGCMs have been evaluated and further improved. However, these metrics are mainly based on variables that are averaged over a given region. In this case, the complexity is reduced, but detailed information from high dimensions is also ignored. As a result, some important aspects of ENSO may be missed. For example, it is not possible to diagnose the internal interactions within the Niño3 region if the Niño3 index is used. For ENSO events with the same amplitude (Niño3 index), it is also difficult to judge whether the events have similar impacts on remote regions without a detailed analysis of how the influences transported. Therefore, to better evaluate the ENSO simulations in CGCMs and further improve our understanding of the dynamics of ENSO, new metrics focusing on more detailed, high dimensional information are needed.

Recently, the concept of complex network has been introduced as a powerful framework for extracting information from large volumes of data, allowing the study of the full complexity of the statistical interdependency structure within a multi-variate dataset^13–31^. In climate science, there are growing interests in applying the concept of complex networks, as climate networks can be easily constructed using different regions (grids) of the world as nodes, and the interactions between the nodes (such as heat, material, or even force exchanges) as links^21^. By measuring the similarity of observations at different nodes, one can determine how nodes are linked in a network, which further allows climate studies from the perspective of complex networks.

In the studies of complex networks, percolation theory is one of the most important findings^32–35^. It indicates the existence of a critical point *P*_*c*_, such that above *P*_*c*_ the phase state of the network may convert abruptly from stable to unstable or metastable (see Methods)^34,35^. This state conversion is named a percolation phase transition, which is an advanced way to measure the non-linearity of a high-dimensional structure. Under the influences of external forces, a percolation phase transition indicates an abrupt collapse of the “old” network. For example, the electrical power networks in the real world can be damaged by malicious attacks or natural disasters, which may further result in abrupt power blackouts^35,36^. In climate science, influences of ENSO on its upper surface air temperature (SAT) network have been recently studied from the perspective of percolation^37–39^. It is found that, as long as the fraction of isolated nodes (nodes with no links with any other node of the network) in the SAT network is higher than a threshold *P*_*c*_ = 0.48, the SAT network will abruptly be divided into many small isolated clusters, indicating a conversion of the network state^37,38^. This abrupt percolation phase transition means that the anomalous SST warming/cooling in the tropical central eastern Pacific has significantly changed the SAT field, which may further transport the influences of ENSO to remote regions via an atmospheric bridge. Accordingly, the fraction of isolated nodes *P* was conjectured to be a new index to determine whether the influences of ENSO can be transported remotely.

Since the abrupt percolation phase transition of the SAT network under the influences of ENSO has been well confirmed using different reanalysis datasets^37^, it can be considered a robust metric with which to evaluate the ENSO simulations in dynamical models. Whether the models are able to capture the percolation phase transition of the SAT network is of great importance for their further simulated ENSO impacts. However, as a newly revealed phenomenon, the current state-of-the-art models may ignore this process and their performance in simulating this phase transition is still unclear. In this work, we aim to address this issue. By studying historical simulations of 15 models participating in phase 5 of the Coupled Model Intercomparison Project (CMIP5), we evaluated their ability in simulating the observed percolation phase transition. From this new perspective, directions for further model improvement are suggested.

The rest of the paper is organized as follows. In the “Results” section, we first study how the simulated SAT networks respond to the anomalous SST warming/cooling in the tropical central eastern Pacific. By comparing the results with those from reanalysis dataset, the performance of the models in simulating the percolation phase transition was evaluated. After listing the models with reasonable simulations and the models with poor simulation skills, we further analyzed the performance from the spatial distribution of simulated node vulnerability and sea surface temperature anomalies (SSTAs) that probably have influences on the performance of percolation phase transition. With all the findings, a detailed discussion is made in the “Discussion and Conclusion” section, and a brief description of the data and methods is provided in a section so-named at the end of this paper.

## Results

### Simulated “percolation phase transition” in CMIP5 models

**Figure 1:**
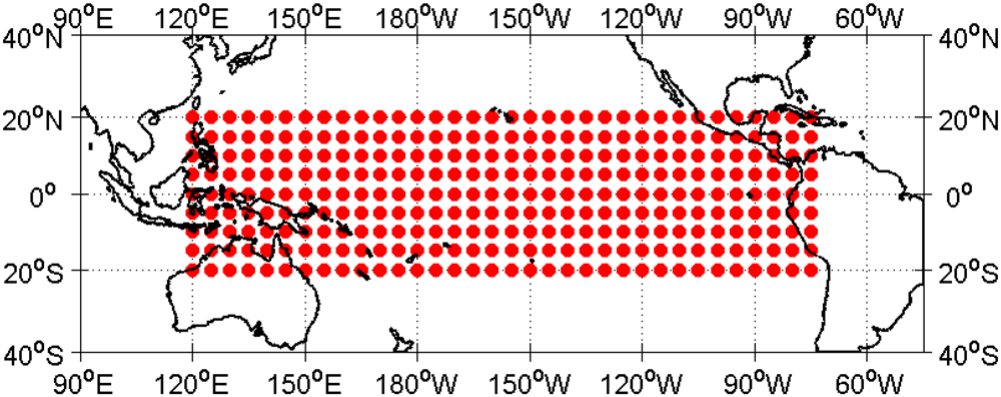
Research domain. In this study, 306 nodes with a resolution of 5° × 5° were selected and the corresponding surface air temperatures were constructed as a climate network. The figure was generated using Matlab (version R2014a, http://www.mathworks.com/pl_homepage).

In this study, we analyzed the historical simulations of 15 CMIP5 models (Table 1). For each model, the surface air temperatures over the tropical Pacific with the domains 120°*E* to 75°*W* and 20°*N* to 20°*S* were constructed as a network with a resolution of 5° × 5° (Fig. 1). To study how the SAT network responds to the anomalous SST warming/cooling in the tropical central eastern Pacific, there are two quantities of interest^38^, as follows:

i) The percentage of isolated nodes *P*. If a node is not connected to any other nodes, we consider it an isolated node. The percentage of isolated nodes *P* is thus defined as the fraction of isolated nodes over the total nodes^40^, which is a quantity that measures the intensity of the influences of ENSO on the upper SAT network (see Methods).
ii) The giant component size *S*. In a network, if there are some nodes that any two of them can be connected with at least one path, we consider these nodes together as a cluster. The giant component size *S* is then defined as the size of the largest cluster divided by the total un-isolated nodes, which is a non-linear measure in percolation theory that can represent the network state (see Methods)^32–35^.

**Table 1:** Information of the CMIP5 models used in this study.

Model	Expansion	Resolution		Length
		Atmos.	Ocean	
NorESM1-M	Norwegian Earth System Model Version 1, Medium Resolution	2.5 × 1.9	1.1 × 0.4	1850–2005
CNRM-CM5	Centre National de Recherches Meteorolog iques, Climate Model Version 5	1.4 × 1.4	1.0 × 0.5	1850–2005
CESM1-FASTCHEM	Community Earth System Model Version 1, Fast Chemistry Mode	1.2 × 0.9	1.1 × 0.4	1920–2005
FGOALS-s2	Flexible Global Ocean-Atmosphere-Land System, Spectral Version 2	2.8 × 1.7	1.0 × 0.8	1850–2005
FGOALS-g2	Flexible Global Ocean-Atmosphere-Land System, Grid-point Version 2	2.8 × 2.8	1.0 × 0.8	1850–2005
GFDL-CM3	Geophysical Fluid Dynamics Laboratory, Climate Model Version 3	2.5 × 2.0	1.0 × 0.8	1860–2005
GFDL-ESM2G	Geophysical Fluid Dynamics Laboratory, Earth System Model Version 2 G	2.5 × 2.0	1.0 × 0.8	1861–2005
HadGEM2-CC	Hadley Global Environment Model Version 2, Carbon Cycle	1.9 × 1.2	1.0 × 0.8	1859–2005
HadGEM2-ES	Hadley Global Environment Model Version 2, Earth System	1.9 × 1.2	1.0 × 0.8	1859–2005
IPSL-CM5B-LR	Institut Pierre-Simon Laplace – Climate Model Version 5B, Low Resolution	3.7 × 1.9	2.0 × 1.0	1850–2005
CMCC-CMS	Centro Euro-Mediterraneo sui Cambiamenti Climatici, Climate Model with a resolved Stratosphere	0.7 × 0.7	2.0 × 1.0	1850–2005
MPI-ESM-P	Max Planck Institute - Earth System Model, Paleo Mode	1.9 × 1.9	0.7 × 0.6	1850–2005
MIROC-ESM	Model for Interdisciplinary Research on Climate, Earth System Model	2.8 × 2.8	1.4 × 0.9	1850–2005
INMCM4	Institute for Numerical Mathematics Climate Model Version 4	2.0 × 1.5	1.0 × 0.5	1850–2005
MRI-ESM1	Meteorological Research Institute, Earth System Model Version 1	1.1 × 1.1	1.0 × 0.4	1851–2005

From left to right, they are: model names, expansions of the model names, resolutions, and the length of the simulation.

**Figure 2:**
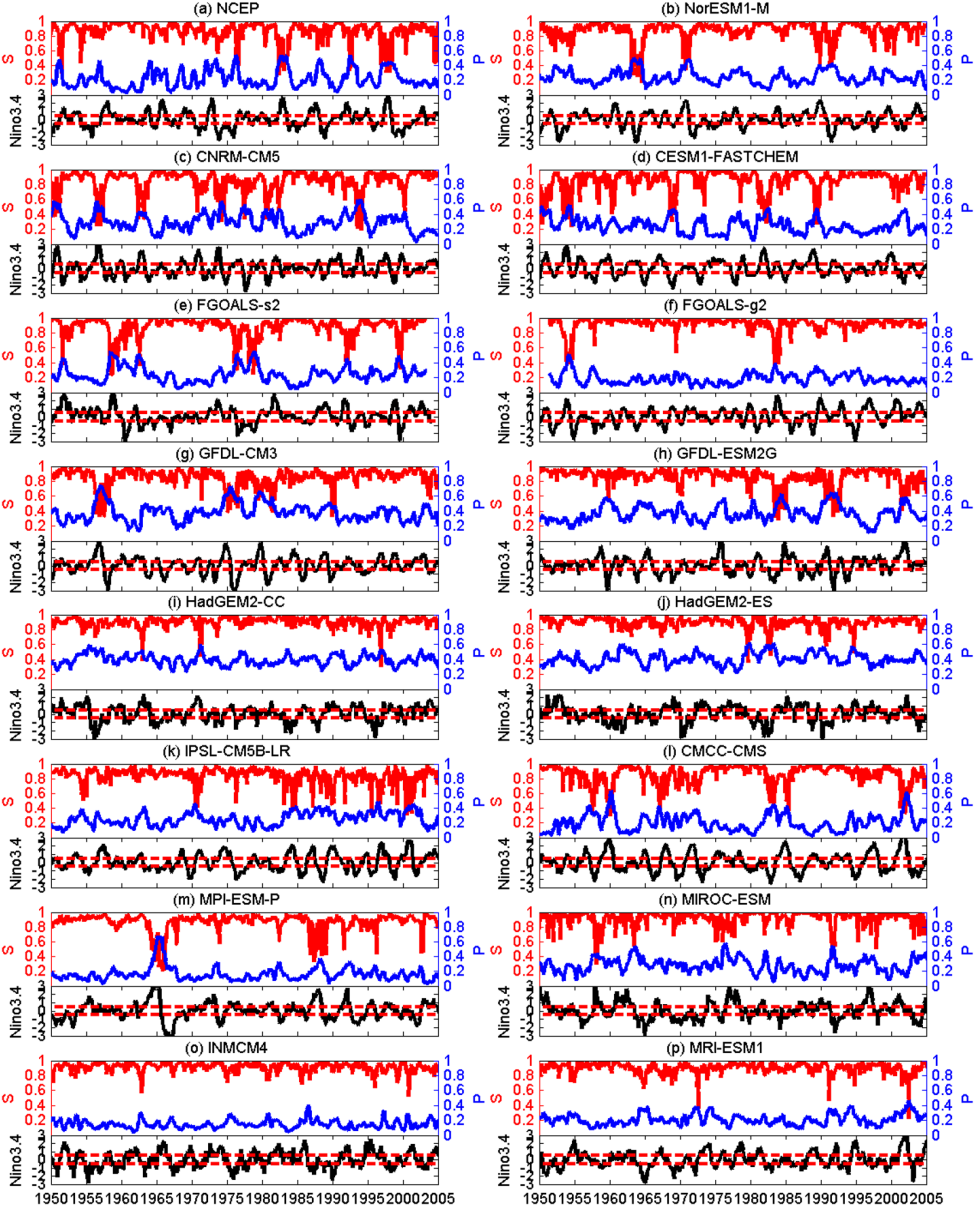
Temporal variation of the giant component size *S* and the percentage of isolated nodes *P*. For each panel, *S* and *P* are represented by red and blue solid lines. The black solid line represents the standardized monthly Niño3.4 index and the red dashed line represents ±0.5. The variables show in Panel (b–p) are based on CMIP5 models datasets, while in Panel (a), the *S* and *P* are calculated from reanalysis datasets.

**Figure 3:**
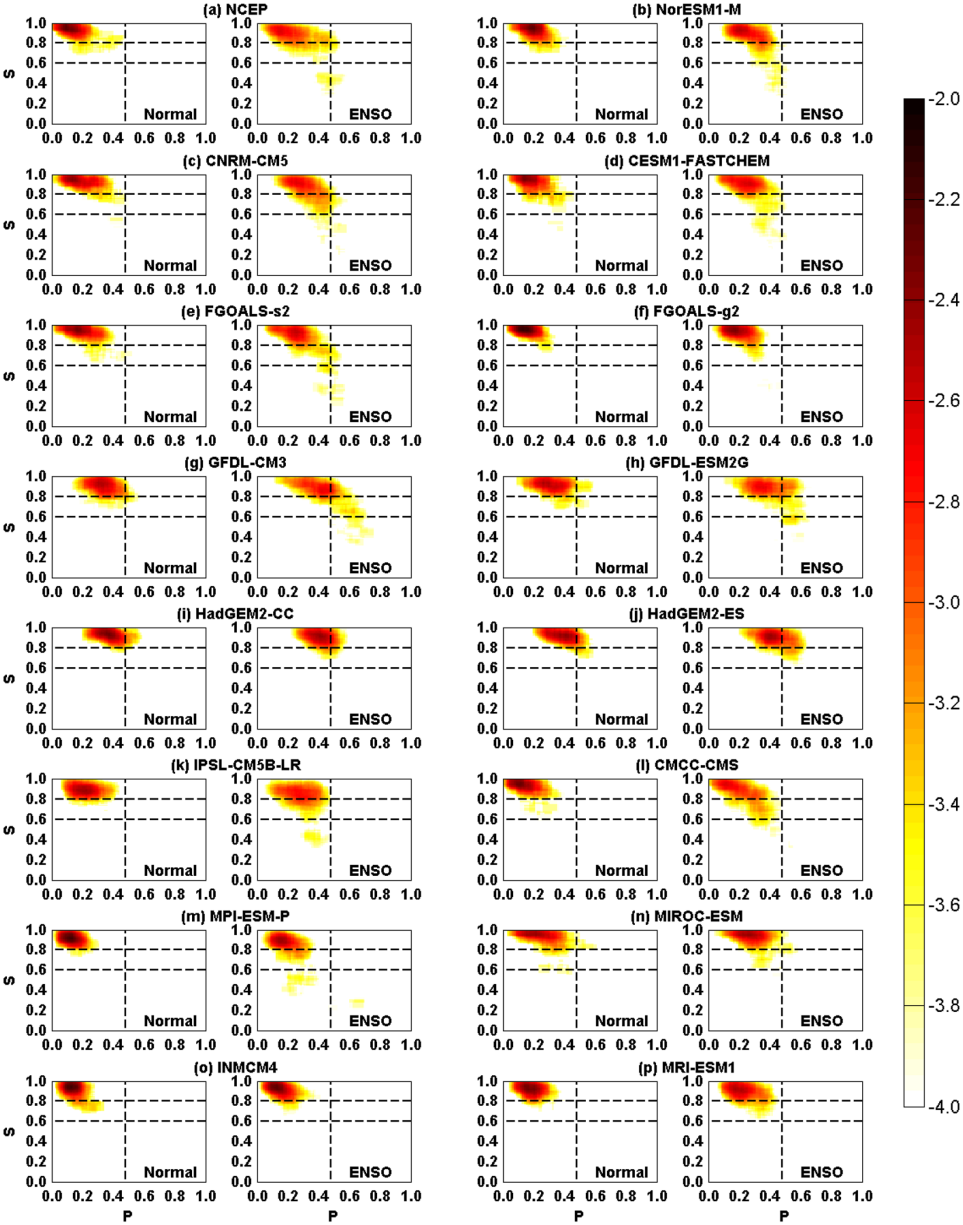
*S* **−** *P* patterns obtained from the NCEP reanalysis data (**a**) and the simulations of the 15 models (**b**–**p**). Two groups (“Normal” and “ENSO”) were classified according to Nino3.4 index. The vertical dashed line represents the percolation threshold (*P*_*c*_ = 0.48) determined from the reanalysis data. The color depicts the probability for a given point of each sub-figure to have a pair of *S* and *P*. The numbers marked in the color bar are transformed by log10. As one can see, most models missed the correct percolation phase transition.

As reported in previous studies, close relations between the two quantities and the Niño3.4 index were found using reanalysis datasets^37,38^. In Fig. 2, we show the giant component size *S* (red) and the percentage of isolated nodes *P* (blue) for each SAT network. From reanalysis data (Fig. 2a), when the standardized monthly Niño3.4 index is larger (smaller) than 0.5 (−0.5), nodes in the upper SAT network are more likely to become isolated (*P* increases) and the giant component size becomes smaller (*S* decreases). In some cases, *S* falls abruptly from a high level to a low level, indicating a phase transition (Fig. 2a)^34,35,37,38,40^. From the model simulations, the anti-correlations between *P* and *S* are well reproduced for nearly all the models (Fig. 2b–p), even though the simulations from some models (CNRM-CM5, IPSL-CM5B-LR, etc.) are more violent, while from some others (INMCM4, MRI-ESM1, etc.) are more stable. However, regarding the percolation phase transitions, different simulations are produced. As shown in Fig. 3, we classified all of the considered time points into two groups according to the definition of an ENSO event. That is, if the standardized three-month running mean Niño3.4 index ≥ (≤) 0.5 (−0.5) for five consecutive overlapping three-month periods, we consider it an El Niño (La Niña) event. Time points in these events are gathered as the “ENSO” group, while other points belong to the “Normal” group. For the “Normal” group, the variations of *S* verus *P* are plotted in the left-hand panel of Fig. 3a–p. In Fig. 3a (reanalysis data), one can see that the giant component sizes *S* of most time points are well above 0.8, implying a well connected network. There is no abrupt changes of *S*. This behavior was well simulated by most models (Fig. 3b–p). Only in the models GFDL-CM3, GFDL-ESM2G, HadGEM2-CC, HadGEM2-ES, and IPSL-CM5B-LR, a rightward shift of the *S* − *P* pattern is found, indicating overestimated *P* values. In the “ENSO” group, however, an abrupt decrease of *S* was observed using the reanalysis data (Fig. 3a). Although the *S* values are higher than 0.8 for most time points during ENSO events, as long as the fraction of isolated nodes reaches the critical points (*P*_*c*_ = 0.48), the *S* drops to a very low level (approximately 0.4), indicating a percolation phase transition. This phenomenon was not reproduced by most models. As in GFDL-CM3, GFDL-ESM2G, and IPSL-CM5B-LR models, the percolation phase transition happens at either larger or smaller *P* values, while for HadGEM2-CC, HadGEM2-ES, INMCM4, and MRI-ESM1, no phase transition is even detected. Only in CNRM-CM5, FGOALS-s2, and NorESM1-M does a reasonable percolation phase transition seem to be reproduced.

Since the “Normal” group gathers the cases in which the SSTA in the tropical central eastern Pacific are moderate, and have limited influences on the upper SAT network^15,20,21,24,37,38^, the simulated *S* − *P* pattern mainly represents the models’ ability to model the atmospheric processes. As shown in the left-hand panel of Fig. 3b–p, most models well reproduced the variations of *S* under different fractions of isolated nodes (*P*). On the contrary, the “ENSO” group represents the cases in which the impacts of SSTA on the upper SAT network are strong^15,20,21,24,37,38^. Accordingly, the *S* − *P* patterns shown in the right-hand panel of Fig. 3b–p mainly indicate the models’ performance in capturing the air-sea interactions, which are found to be poor even by visual inspection of Fig. 3.

**Figure 4:**
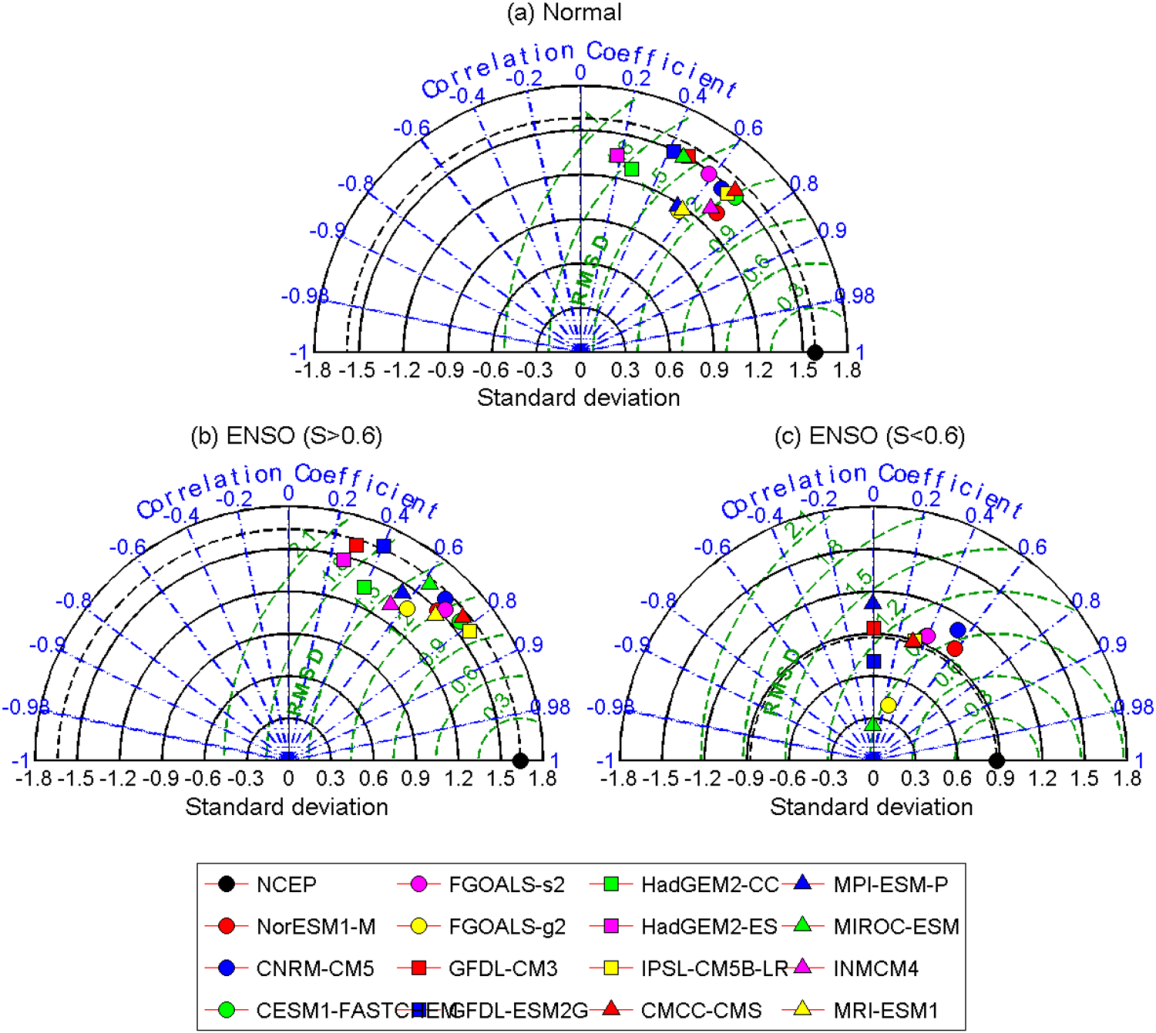
Taylor diagram of *S* − *P* patterns shown in Fig. 3. (**a**) Shows the results for the “Normal” group, (**b**) for the “ENSO (*S* > 0.6)” group, while (**c**) the “ENSO (*S* < 0.6)” group. Each dot represents a model as shown in the bottom box, while the black dot stands for the results calculated from NCEP reanalysis data, which serves as the reference. The azimuthal positions represent the spatial correlation coefficients between the *S* − *P* pattern obtained from the reanalysis data and those simulated from models. The radial distance from origin to each dot stands for the standard deviation, and the distances from the black dot to the other dots are the root mean square errors (RMSE). It is worth to note that, since the percolation phase transition is missed by HadGEM2-CC, HadGEM2-ES, INMCM4, and MRI-ESM1, no dots are shown for the four models in (**c**).

It is worth noting that not all the time points within an ENSO event point to low *S* values. The percolation phase transition does not occur with the arising of an ENSO event. Only when the fraction of isolated nodes reaches 48% do the *S* values decrease from a higher level (*S* > 0.6, above 0.8 for most cases) to a lower level (*S* < 0.6, approximately 0.4 for most cases). This means that even if the SAT network is influenced by the SSTA below, as long as the impacts are not strong enough, a percolation phase transition cannot be triggered. Accordingly, we further divided the “ENSO” group into two parts: one includes the cases when the percolation phase transition is not triggered (*S* > 0.6), and the other is for the time points at which the percolation phase transition occurred (*S* < 0.6). To better evaluate the models’ ability to simulate the percolation phase transition, we further presented the Taylor diagrams^11,41^ for the “Normal” group, the “ENSO” group with *S* > 0.6, and the “ENSO” group with *S* < 0.6 separately. As shown in Fig. 4, results from the reanalysis dataset are considered as the “reference” (black point in Fig. 4), while results from models are marked in the diagram with other colors. The cosine of the polar angle represents the spatial correlation coefficient (SCC) between the observed (reanalysis dataset) and the modeled *S* − *P* patterns^41^. The distance from each point to the origin is the standard deviation (SD), while the distance to the “reference” point is the RMSE^41^. In general, the SCC refers to phase simulation while standard deviation refers to amplitude simulation. Thus, a model simulation that is relatively accurate would lie near the dotted arc (indicating similar variance) and close to the reference point. From Fig. 4a, it is clear that the *S* − *P* patterns simulated by more than half of the models have similar SD to that calculated from the reanalysis dataset. As for the SCC, except the GFDL-CM3, GFDL-ESM2G, HadGEM2-CC, HadGEM2-ES, and MIROC-ESM models, all of the other models have high SCCs between 0.5 and 0.8 (see Fig. 4). Accordingly, most models can reproduce the *S* − *P* pattern for the “Normal” group with small RMSE. For the *S* − *P* patterns in “ENSO” groups with *S* > 0.6, similar results are found as in the “Normal” groups (Fig. 4b). Most models have reasonable simulations of the *S* − *P* pattern with similar SD and high SCC (0.6–0.8). However, regarding the “ENSO” groups with *S* < 0.6 (Fig. 4c), poor simulation skills of the models are found. In addition to the four models without percolation phase transition (HadGEM2-CC, HadGEM2-ES, INMCM4, and MRI-ESM1; see Fig. 3), there are another five models (MIROC-ESM, GFDL-CM3, GFDL-ESM2G, FGOALS-g2, and MPI-ESM-P) in which the simulated *S* − *P* patterns are nearly uncorrelated with the “reference”. Moreover, the SCCs are also very low in the IPSL-CM5B-LR, CMCC-CMS, CESM1-FASTCHEM and FGOALS-s2 models. Only two models (NorESM1-M and CNRM-CM5) show some skills in simulating the *S* − *P* pattern. They have relatively high SCC values (0.5–0.6), reasonable SDs, and most importantly, smaller RMSEs. From Fig. 4c, the NorESM1-M model seems to provide the best simulations of the percolation phase transition.

**Figure 5:**
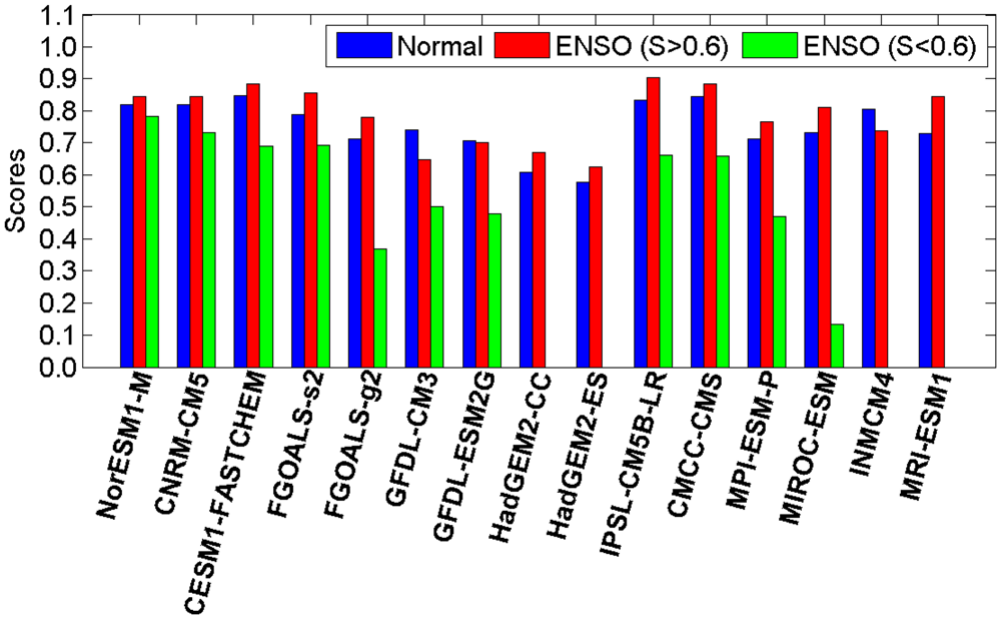
Skill scores of the 15 models on simulating the *S* **−** *P* patterns in “Normal” group (blue bars), “ENSO” group with *S* > 0.6 (red bars), and “ENSO” group with *S* < 0.6 (green bars). The specific values of the skill scores are listed in Table 2.

To quantify the models’ performance in simulating the *S* − *P* patterns, we further calculated the skill scores as defined by^41^:

1 $$H=\frac{\mathrm{4(1}+R)}{{({\hat{\sigma }}_{f}+\mathrm{1/}{\hat{\sigma }}_{f})}^{2}\mathrm{(1}+{R}_{0})},$$

where *R* is the spatial correlation coefficient, ${\hat{\sigma }}_{f}$ the spatial standard deviation of the simulated *S* − *P* pattern divided by that of the observed *S* − *P* pattern, and *R*_0_ the maximum correlation attainable^42^. Here, we assumed that *R*_0_ = 1. As shown in Fig. 5 and Table 2, high skill scores are found in the “Normal” group. There are six models with skill scores of *H* ≥ 0.8, and nearly all the models (13 out of 15) have scores higher than 0.7. As discussed above, these high skill scores indicate reasonable abilities of the current models to simulate atmospheric processes. In other words, the percolation properties of the SAT network can be well modeled if the atmospheric field is not influenced by the SST anomalies below. As for the “ENSO” group with *S* > 0.6, for most models, the skill scores are close to those obtained from the “Normal” group. There are eight models with skill scores higher than 0.8, and the scores of all 15 models are higher than 0.6. Therefore, it is evident to speculate that, as long as the ENSO impacts are not strong enough to trigger a phase transition, the current models have the ability to simulate the percolation properties of the SAT network. However, regarding the “ENSO” group with *S* < 0.6, which represents the cases when the percolation phase transitions are triggered, much lower scores are obtained for all the models. As shown in Fig. 5 and Table 2, only four models (NorESM1-M, CNRM-CM5, FGOALS-s2 and CESM1-FASTCHEM) have scores higher than 0.7, but nine models have scores no higher than 0.5. Combined with the simulated *S* − *P* patterns shown in Fig. 3, we can only cautiously recommend four models (NorESM1-M, CNRM-CM5, FGOALS-s2 and CESM1-FASTCHEM) that have certain abilities to simulate the percolation phase transition. In other words, it is still challenging to fully capture the sea-air interactions over the Pacific. From the perspective of percolation, most models failed in properly simulating the responses of the SAT field to the anomalous SST warming/cooling.

**Table 2:** Skill scores of the 15 models on simulating the *S* − *P* patterns in “Normal” group, “ENSO” group with *S* > 0.6, and “ENSO” group with *S* < 0.6.

Rank	Normal		ENSO (*S* > 0.6)		ENSO (*S* < 0.6)	
01	CESM1-FASTCHEM	0.85	IPSL-CM5B-LR	0.90	NorESM1-M	0.78
02	CMCC-CMS	0.84	CESM1-FASTCHEM	0.89	CNRM-CM5	0.73
03	IPSL-CM5B-LR	0.83	CMCC-CMS	0.89	FGOALS-s2	0.70
04	NorESM1-M	0.82	FGOALS-s2	0.86	CESM1-FASTCHEM	0.70
05	CNRM-CM5	0.82	CNRM-CM5	0.85	IPSL-CM5B-LR	0.66
06	INMCM4	0.80	NorESM1-M	0.84	CMCC-CMS	0.66
07	FGOALS-s2	0.79	MRI-ESM1	0.84	GFDL-CM3	0.50
08	GFDL-CM3	0.74	MIROC-ESM	0.81	GFDL-ESM2G	0.48
09	MIROC-ESM	0.73	FGOALS-g2	0.78	MPI-ESM-P	0.47
10	MRI-ESM1	0.72	MPI-ESM-P	0.77	FGOALS-g2	0.37
11	FGOALS-g2	0.71	INMCM4	0.74	MIROC-ESM	0.13
12	MPI-ESM-P	0.71	GFDL-ESM2G	0.70	HadGEM2-CC	0.00
13	GFDL-ESM2G	0.71	HadGEM2-CC	0.67	HadGEM2-ES	0.00
14	HadGEM2-CC	0.61	GFDL-CM3	0.65	INMCM4	0.00
15	HadGEM2-ES	0.58	HadGEM2-ES	0.62	MRI-ESM1	0.00

### “Node vulnerability” and “SSTA” composites in CMIP5 models

**Figure 6:**
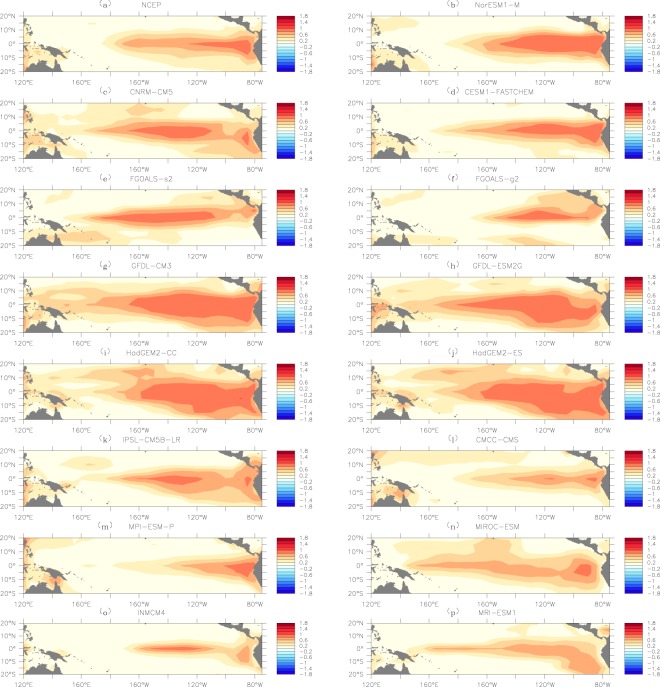
Spatial distributions of node vulnerabilities *F*_*i*_ in the SAT network. (**a**) Shows the results from NCEP reanalysis data, while (**b**–**p**) shows the simulated results from the 15 models. Nodes with higher frequency (chance) of being isolated are marked by dark color. This figure is generated using Ferret (version6.9, http://ferret.pmel.noaa.gov/Ferret/documentation/release-notes/version-6-9-release-notes).

Since the giant component size *S* is closely related to the node-connection pattern of a given network, to study why some models (such as NorESM1-M) reproduced the observed *S* − *P* pattern while some others (such as INMCM4, MRI-ESM1, etc.) failed, it is straightforward to investigate the node vulnerability *F*_*i*_ in the network. As described in the “Methods” section, *F*_*i*_ is a quantity that measures how vulnerable a node is when the network is influenced^37,38^. It is defined as the ratio of the times that a given node is isolated to the entire time period of an ENSO event. If the ratio is high, we consider the node to have high vulnerability. Based on reanalysis data (Fig. 6a), it has been found that nodes in the SAT network are highly vulnerable over the tropical central eastern Pacific. This is reasonable since ENSO events have the strongest influences on the upper SATs over this key region^1,43,44^. Consequently, node links in this region are easy broken and the nodes are more likely to be isolated^15,20,21^. From model simulations, we find that models with good skills in simulating the percolation phase transition (e.g., NorESM1-M) have similar *F*_*i*_ patterns as that obtained from reanalysis data. However, those that cannot reproduce the desired phase transition (e.g., INMCM4, GFDL-CM3, etc.) fail in fully capturing the observed *F*_*i*_ pattern. For instance, one can see clearly that the simulated vulnerable areas determined by GDFL-CM3 (Fig. 6g) and GFDL-ESM2G (Fig. 6h) are much larger than that obtained from reanalysis data. This overestimated node vulnerability may lead to a percolation phase transition, but at larger *P* values (Fig. 3g,h). For models with smaller vulnerable areas (e.g., INMCM4 and MRI-ESM1), the node links are too stable. This underestimated node vulnerability may be responsible for missing the percolation phase transition (Fig. 3o,p). For other models such as FGOALS-g2, HadGEM2-CC, HadGEM2-ES, IPSL-CM5B-LR, MPI-ESM-P, and MIROC-ESM, the simulated *F*_*i*_ distributions are remarkably different from the observed pattern, which may contribute to the biased simulations of the percolation phase transition.

In addition to the tropical central eastern Pacific, one may also note the fairly strong node vulnerabilities *F*_*i*_ in the far western Pacific (Fig. 6a). Since the far western Pacific is a key region for the generation of ENSO events, i.e. precursory ocean-atmosphere signals (such as the anomalous oceanic thermocline depth signal^45^ and the westerly wind events in the atmosphere^46–48^) that could trigger an ENSO event usually occur in the far western Pacific^49–51^, the strong *F*_*i*_ values in this region may have physical meanings. As shown in Fig. 6c,d, some models with certain abilities to simulate the percolation phase transition also reproduced strong *F*_*i*_ values in the far western Pacific. While for some models with poor performance in simulating the percolation phase transition, the *F*_*i*_ patterns in the far western Pacific were not fully captured. These findings suggest that the far western Pacific may be also important for the simulations of the percolation phase transition in the SAT network.

**Figure 7:**
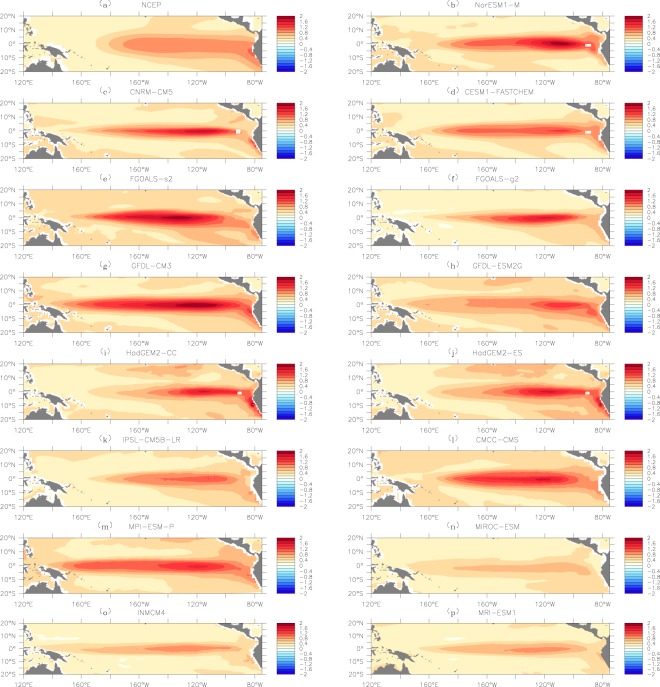
Spatial distributions of the sea surface temperature (SST) anomalies. Similar to Fig. 6, (**a**) shows the results from NCEP reanalysis data, while (**b**–**p**) shows the simulated results from the 15 models. The absolute SST anomalies were calculated as the composites of both the positive anomalies and negative anomalies (taking the absolute values). Nodes with stronger absolute SST anomalies are marked by dark red color. The figure is generated using Ferret (version6.9, http://ferret.pmel.noaa.gov/Ferret/documentation/release-notes/version-6-9-release-notes).

Since the node vulnerability in the SAT network is related to the SST anomalies^37^, the simulated SSTA might give an in-depth understanding of why the percolation phase transition differs greatly among different models. Especially considering that percolation phase transition measures the models’ ability to capture sea-air interactions, it is essential to further compare the simulated SSTA with the node vulnerability in the upper SAT network. As shown in Fig. 7, since the node vulnerability is related to the magnitude of SSTA, the composites of SSTA during ENSO events (La Niña events being represented by the absolute value) are calculated. From reanalysis data (Fig. 7a), one can see that the regions with strong absolute SSTAs are exactly the same regions with high *F*_*i*_ (Fig. 6a). This is reasonable as the SSTA should have the strongest influences on the upper SAT of the same region^1,43,44^. In other words, if the sea-air interaction is fully captured, the *F*_*i*_ patterns in the tropical central eastern Pacific should be similar to the SSTA patterns in Fig. 7. However, by comparing the SSTA patterns in Fig. 7 with the *F*_*i*_ patterns in Fig. 6, we found mismatches in many models. For example, in the GFDL-CM3 and MIROC-ESM models, the vulnerable regions with high *F*_*i*_ (Fig. 6g,n) are much larger than the regions with strong SSTA (Fig. 7g,h and n). In the FGOALS-g2, MPI-ESM-P, and INMCM4 models, the simulated vulnerable regions in the tropical central eastern Pacific (Fig. 6f,m and o) are smaller than the regions with strong SSTA (Fig. 7f,m and o). All these models with mismatches between the SSTA patterns and the *F*_*i*_ patterns in the tropical central eastern Pacific fail in reproducing the desired percolation phase transition. Only in the models in which the two patterns in Figs 6 and 7 are similar, such as NorESM1-M, was the percolation phase transition reasonably simulated. Accordingly, the improper simulations of percolation phase transition may rely on the mismatches of the SSTA pattern and the *F*_*i*_ pattern in the tropical central eastern Pacific, or in other words, the imperfectly simulated sea-air interactions.

## Discussion and Conclusions

In this study, we evaluated the ability of 15 CMIP5 models to simulate ENSO from a new perspective, namely as a climate network. By constructing the upper SAT as a network and studying its percolation properties under different SST conditions, we found that most models can simulate the percolation properties of the SAT network successfully at weak SST anomalies, but fail to correctly simulate the percolation phase transition when the SST anomalies were strong enough to trigger a state change of the SAT network. For the 15 models, four models missed the observed percolation phase transition, seven models reproduced the percolation phase transition at incorrect critical point *P*_*c*_. Only four models (NorESM1-M, CNRM-CM5, FGOALS-s2, and CESM1-FASTCHEM) seemed to be able to capture the desired percolation phase transition, but their skill scores calculated from Taylor diagrams were relatively low. As the percolation property essentially measures the fragmentation and functionality of a network, its phase transition indicates a state conversion^40^. Therefore, whether the models simulated the percolation phase transition of the SAT network successfully is related to the models’ ability to fully capture the influences of SSTA on the upper SAT field, or, in other words, the sea-air interaction. In this work, we find most models fail to fully capture the sea-air interactions. By comparing Figs 6 with 7, it is found that for most models the regions with strong absolute SSTA are different from the regions with high node vulnerability in the SAT network. Only a few models, such as NorESM1-M and FGOALS-s2, produced similar spatial patterns of the node vulnerability (Fig. 6) and the SSTA (Fig. 7). This inconsistency implies the improper simulations of the percolation phase transition may rely on the mismatches of the SSTA pattern and the *F*_*i*_ pattern in the tropical central eastern Pacific, which suggests a new direction for further improvement of dynamical models.

It is worth noting that when studying the simulated percolation properties, we used the simulated Niño3.4 index to classify both the “Normal” and “ENSO” groups. Therefore, the low skills of most models in simulating the percolation phase transition are not related to the models’ ability to simulate the SST anomalies. More likely, it is the inappropriate atmospheric component, or the coupler of the CMIP5 models that leads to the poor simulations. As discussed in^37^, it has been proposed that the differences in performance between the FGOALS-s2 and FGOALS-g2 models in simulating the percolation phase transition mainly arise from the different atmospheric components. In this study, the simulations of these two models show good agreement with the previous study, although the data length is slightly different between the two works^37^. However, for the other two pairs of models that are the different versions of the same model, GFDL-CM3/GFDL-ESM2G and HadGEM2-CC/HadGEM2-ES, significantly different simulation performance is not shown. In fact, both versions of the HadGEM2 model (HadGEM2-CC and HadGEM2-ES) miss the percolation phase transition (Fig. 3), while both versions of the GFDL model (GFDL-CM3 and GFDL-ESM2G) fail in simulating the transition at the correct *P*_*c*_ value. Therefore, more studies are required to improve these models’ ability to simulate sea-air interactions.

Finally, we emphasize that the percolation phase transition is not only a metric for the evaluation of ENSO simulations. It may also serve as an indicator for the ENSO impacts. In previous studies^37,38^, it has been conjectured that the transition of the percolation phase indicates that the upper SAT field has been fully changed by the SSTA, and the influences of ENSO may be further transported to remote regions through an atmospheric bridge^52,53^. If there is no percolation phase transition, however, the influences of ENSO may be mainly trapped in nearby regions. In this study, we can see that even in the “ENSO” group there are also stages in which the phase state stays stable with *S* > 0.6. This indicates that despite a ENSO event arises, the SAT network may still stay stable until the percentage of isolated nodes exceeds the threshold *P*_*c*_. Accordingly, compared to the Niño3.4 index, checking the percolation properties of the SAT network maybe a better way to judge the ENSO impacts. Although this discussion is beyond the scope of the present work, it deserves more detailed study in the future.

## Data and Methods

### Data

In this study, historical simulations of 15 CMIP5 models were analyzed. Information on the 15 models is listed in Table 1. For each model, the simulated daily SAT and monthly SST data were downloaded from the Earth System Grid Federation (ESGF) via https://esgf-data.dkrz.de/search/cmip5-dkrz/. All of the data end in 2005 and cover at least 100 years. Using SAT data, the SAT network was constructed over the domain 120*°E* to 75°*W* and 20°*N* to 20^o^*S* (Fig. 1). Standardized monthly Niño3.4 indices were calculated from SST. In addition to the model simulated data, we also downloaded daily SAT data from the NCEP/NCAR reanalysis 1 project at the National Oceanic & Atmospheric Administration (NOAA, provided by the NOAA/OAR/ESRL PSD, Boulder, Colorado, USA, via http://www.esrl.noaa.gov/psd/data/gridded/data.ncep.reanalysis.surface.html), and the monthly Niño3.4 index from NOAA Extended Reconstructed SST V5 via http://www.esrl.noaa.gov/psd/data/climateindices/^54^. The reanalysis SAT data were used to construct a SAT network. Since it covers only 69 years (1948–2016) with a spatial resolution of 2.5° × 2.5°, to maintain consistency among different datasets, the SAT networks were constructed using data from 1950 to 2005 with the spatial resolution set as 5° × 5°. As for the standardized three-month running mean Niño3.4 indices, we defined an ENSO event when the indices ≥(≤) +0.5(−0.5) for five consecutive month periods. Monthly SST anomalies (with annual cycle removed) were also used in this study (see Fig. 7). The data were downloaded from NOAA via https://www.esrl.noaa.gov/psd/data/gridded/data.noaa.ersst.v4.html.

## Methods

### Surface air temperature Network

In this study, we employed the nonlinear synchronization measure to construct a SAT network. As shown in Fig. 1, each node is marked with numbers from 1 to 306 as node indexes according to the sequence from west to east and from north to south. For the SAT in each node, we first calculated the anomalies by subtracting the long-term mean annual cycle *T*_*k*_(*d*), where *k* represents the node index (1-306) and *d* is the calendar date. For every 30th day *t* in the considered time span between January 1950 and August 2005, we then computed the time-delayed cross-correlations for each pair of nodes *i* and *j* over 365 days before *t*, with time lags *τ* between −200 days and 200 days. The coefficient is denoted as ${C}_{i,j}^{t}(\tau )$. For each time point *t*, the link strength between nodes *i* and *j* can thus be defined as^15,21^:

2 $${W}_{i,j}^{t}=\frac{max(|{C}_{i,j}^{t}(\tau )|)-mean(|{C}_{i,j}^{t}(\tau )|)}{std(|{C}_{i,j}^{t}(\tau )|)}\mathrm{.}$$

A pair of nodes is considered connected if their link strength is above a threshold *Q*. In view of systematic biases between different models, it is necessary to standardize link strength ${W}_{i,j}^{t}$ so that the biases could be eliminated. Here, we determined a threshold *Q* = 3.1 for the confidence level of 99% (see Supplementary Materials, Fig. S1). Using the Heaviside function, we represented this definition as:

3 $${A}_{i,j}^{t}=\theta ({W}_{i,j}^{t}-Q)=\{\begin{array}{ll}\mathrm{1,} & {W}_{i,j}^{t} > Q\\ \mathrm{0,} & {W}_{i,j}^{t} < Q\end{array},$$

and the degree of node *i* at time *t* as^37,38^:

4 $${K}_{i}^{t}=\sum _{j=1}^{j=306}\,{A}_{i,j}^{t}\mathrm{.}$$

If at a given time point *t*, node *i* has no connection with any other nodes, i.e., ${K}_{i}^{t}=0$, we designated it an isolated node. The isolated nodes can be counted by a new quantity^37,38^:

5 $${R}_{i}^{t}=\{\begin{array}{ll}\mathrm{1,} & {K}_{i}^{t}=0\\ \mathrm{0,} & {K}_{i}^{t} > 0\end{array},$$

where *i* is the node index from 1 to 306.

### Percentage of isolated nodes

In the SAT network, the isolated nodes can be considered as the result of influences from ENSO events. Accordingly, for each time point *t*, we defined the intensity of influences as^37,38,55^:

6 $${P}^{t}=\frac{\sum _{i=1}^{i=306}\,{R}_{i}^{t}}{306},$$

where *P*^*t*^ denotes the fraction of the isolated nodes at time point *t* (see Fig. 2).

### Node Vulnerability

To quantify the chance of a node to be isolated under influences, we defined another quantity as^37,38^:

7 $${F}_{i}=\frac{\sum _{t\in T}\,{R}_{i}^{t}}{L(T)},$$

where *L*(*T*) is the length of a given time period (or the number of the total time points), and *F*_*i*_ is the fraction of the time points when node *i* is isolated (see Fig. 6).

### Giant component size

Giant component size is used to indicate the phase state of a network and measure its fragmentation and functionality. In a network, if there are some nodes such that any two of them can be connected with at least one path, we consider these nodes together as a cluster. By definition, there is no isolated node in a cluster. By counting the number of the nodes, cluster size is quantified. For the cluster with the largest size (highest number of nodes), we define it as the largest cluster. The giant component size *S* can then be defined as^37,38,55^:

8 $${S}^{t}=\frac{{N}_{LC}^{t}}{306-\sum _{i=1}^{i=306}\,{R}_{i}^{t}},$$

where ${N}_{LC}^{t}$ is the number of nodes in the largest cluster, and *S*^*t*^ represents the giant component size at time point *t* (see Fig. 2). According to *S*, the phase state of a given network could be theoretically classified as stable (*S* = 1), unstable (*S* → 0), or metastable (0 < *S* < 1)^32–35^. However, in practice, it is difficult to find a full stable network with *S* = 1, and an unstable network with *S* → 0. Therefore, the concepts of stable/unstable are normally discussed relatively. As in our study, the phase state of a SAT network is considered relatively stable when *S* is around 0.8 (Fig. 3a, left-hand panel), while it is considered metastable or relatively unstable when *S* is around 0.4 (Fig. 3a, right-hand panel).

## Electronic supplementary material

Supplementary materials
