# Supplementary material for: Evaluation of ENSO simulations in CMIP5 models: A new perspective based on percolation phase transition in complex networks
Source: Sci Rep. 2018 Oct 8;8:14912. doi: 10.1038/s41598-018-33340-y (PMC6175830; doi:10.1038/s41598-018-33340-y)
Supplement: Supplementary file 1 — Supplementary materials [file 41598_2018_33340_MOESM1_ESM.pdf]

Evaluation of ENSO simulations in CMIP5 models: A new perspective based on percolation phase transition in complex networks

(Supplementary Materials)

Zhenghui Lu[1,2], Zuntao Fu[2]*, Lijuan Hua[3], Naiming Yuan[1]†, Lin Chen[4]

1 CAS Key Laboratory of Regional Climate Environment for Temperate East Asia,

Institute of Atmospheric Physics, Chinese Academy of Sciences, 100029, Beijing, China

2 Lab for Climate and Ocean-Atmosphere Studies, Dept. of Atmospheric and Oceanic Sciences,

School of Physics, Peking University, Beijing, 100871, China

3 State Key Laboratory of Severe Weather (LASW),

Chinese Academy of Meteorological Sciences, Beijing 100081, China

4 International Pacific Research Center, and School of Ocean and Earth Science and Technology,

University of Hawaii at Manoa, Honolulu, Hawaii, USA

*Correspondence and requests for materials can be addressed to Z.F. Email: fuzt@pku.edu.cn

†Correspondence and requests for materials can also be addressed to N.Y. Email: naimingyuan@hotmail.com

Supplementary materials for determining connections between nodes. According to Eq. (1) in the main text, one can always calculate a link strength $W^t_{i,j}$ between node i and j, at time point t. But whether the link has true physical meanings, it is still unclear and one has to further determine a threshold Q [1, 2, 3]. In this study, we followed the previous study [1] and determined the threshold Q by shuffling the original time series at each node for $1,000$ times. After calculating the corresponding link strengths $W^t_{s;i,j}$, we are able to compare the probability density functions (PDF) of the link strengths $W^t_{s;i,j}$ with that obtained from $W^t_{i,j}$. As shown in Fig. S1, at the significance level of 0.01, we determined the threshold $Q = 3.1$, above which a true connection between i and j can be confirmed. It is worth to note that, since different models have different system biases, we standardized the link strength respectively for each network before estimating the PDF. This is different from the previous work [1].

References

[1] Lu, Z., Yuan, N., and Fu, Z. Percolation Phase Transition of Surface Air Temperature Networks under Attacks of El Niño/La Niña, *Sci. Rep.*, **6**, 26779 (2016).

[2] Hua, L. et al. Percolation Phase Transition of Surface Air Temperature Networks: A new test bed for El Niño/La Niña simulations, *Sci. Rep.*, **7**, 8324 (2017).

[3] Radebach, A., Donner, R. V., Runge, J., Donges, J. E., and Kurths, J. Disentangling different types of El Niño episodes by evolving climate network analysis, *Phys. Rev. E*, **88**, 052807 (2013).

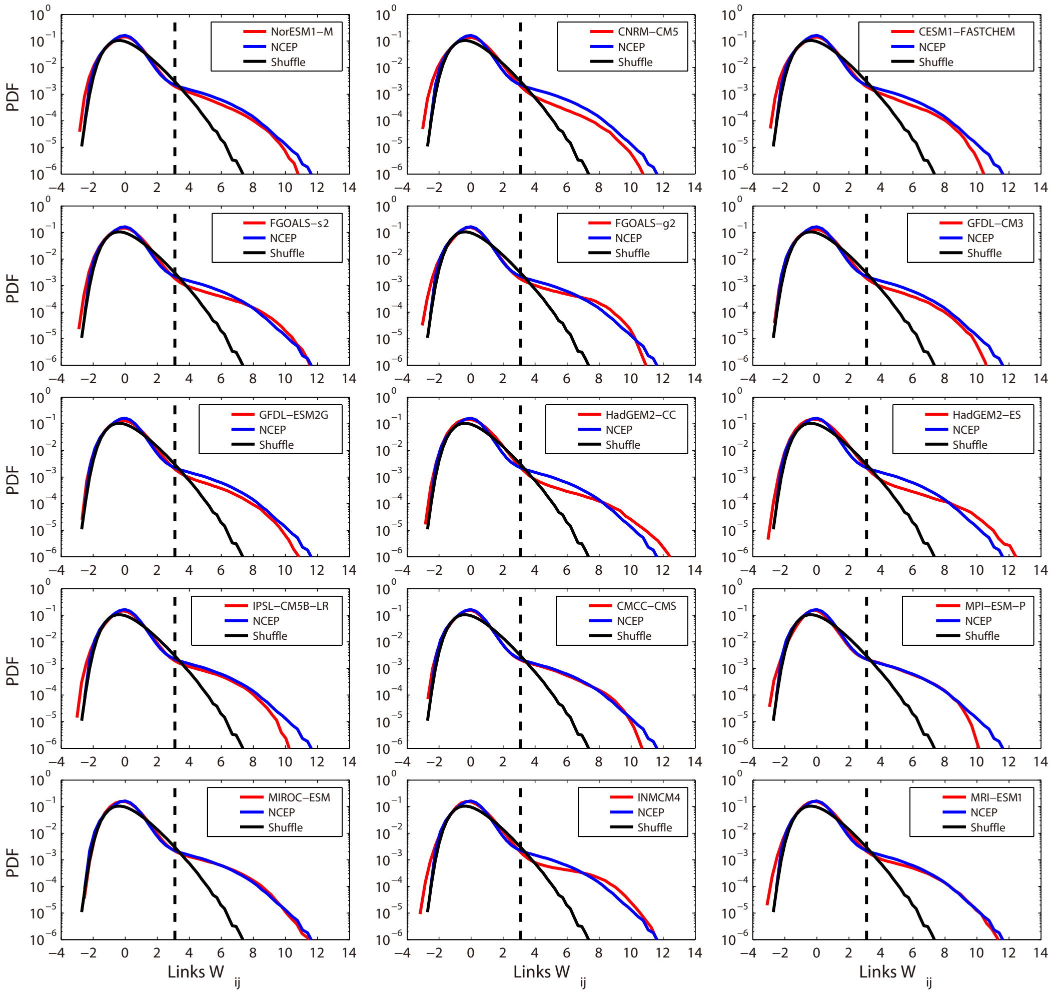

Supplementary Figure 1: **Probability density functions (PDF) of link strengths.** The red lines in these 15 sub-figures represent the PDFs of link strengths calculated from the simulations of the 15 CMIP5 models, the blue lines show the results calculated from NCEP reanalysis dataset, while the black lines are for the randomly shuffled network. The dashed line shows the threshold of 3.1, which represents the significance level of 0.01. In order to avoid the influences of model system bias, all the links for each case have been standardized respectively, before estimating the PDFs.
